# Methamphetamine and the Synthetic Cathinone 3,4-Methylenedioxypyrovalerone (MDPV) Produce Persistent Effects on Prefrontal and Striatal Microglial Morphology and Neuroimmune Signaling Following Repeated Binge-like Intake in Male and Female Rats

**DOI:** 10.3390/brainsci14050435

**Published:** 2024-04-27

**Authors:** Erin K. Nagy, Paula F. Overby, Jonna M. Leyrer-Jackson, Vincent F. Carfagno, Amanda M. Acuña, M. Foster Olive

**Affiliations:** 1 Department of Psychology, Behavioral Neuroscience and Comparative Psychology Area, Arizona State University, Tempe, AZ 85287, USA; 2 Department of Medical Education, School of Medicine, Creighton University, Phoenix, AZ 85012, USA; 3 Arizona College of Osteopathic Medicine, Midwestern University, Glendale, AZ 85308, USA; 4 Interdisciplinary Graduate Program in Neuroscience, School of Life Sciences, Arizona State University, Tempe, AZ 85287, USA

**Keywords:** methamphetamine, methylenedioxypyrovalerone, binge intake, prefrontal cortex, striatum, neuroinflammation, cytokine, microglia

## Abstract

Psychostimulants alter cellular morphology and activate neuroimmune signaling in a number of brain regions, yet few prior studies have investigated their persistence beyond acute abstinence or following high levels of voluntary drug intake. In this study, we examined the effects of the repeated binge-like self-administration (96 h/week for 3 weeks) of methamphetamine (METH) and 21 days of abstinence in female and male rats on changes in cell density, morphology, and cytokine levels in two addiction-related brain regions—the prefrontal cortex (PFC) and dorsal striatum (DStr). We also examined the effects of similar patterns of intake of the cocaine-like synthetic cathinone derivative 3,4-methylenedioxypyrovalerone (MDPV) or saline as a control. Robust levels of METH and MDPV intake (~500–1000 infusions per 96 h period) were observed in both sexes. We observed no changes in astrocyte or neuron density in either region, but decreases in dendritic spine densities were observed in PFC pyramidal and DStr medium spiny neurons. The microglial cell density was decreased in the PFC of METH self-administering animals, accompanied by evidence of microglial apoptosis. Changes in microglial morphology (e.g., decreased territorial volume and ramification and increased cell soma volume) were also observed, indicative of an inflammatory-like state. Multiplex analyses of PFC and DStr cytokine content revealed elevated levels of various interleukins and chemokines only in METH self-administering animals, with region- and sex-dependent effects. Our findings suggest that voluntary binge-like METH or MDPV intake induces similar cellular perturbations in the brain, but they are divergent neuroimmune responses that persist beyond the initial abstinence phase.

## 1. Introduction

Chronic exposure to or intake of psychostimulants such as methamphetamine (METH) can alter the density and morphology of various cell types in the brain [1,2]. These effects are accompanied, and perhaps mediated, by neuroimmune signaling [3,4,5,6]. Such signaling can be initiated and sustained by one or more of a multitude of processes, including peripheral immune cell infiltration into the brain, direct central activation of key immune signaling mediators, stimulation of neuron–glia cytokine signaling, and overproduction of cytotoxic reactive nitrogen or oxygen species. Neuroinflammatory signaling can perturb brain function and may influence some features of addiction, such as poor impulse control, cognitive inflexibility, maladaptive learning and memory, and loss of sensitivity to negative consequences [7,8,9]. More recently, it has become evident that novel psychostimulants, including synthetic cathinone derivatives such as 4-methylmethcathinone (mephedrone) and 3,4-methylenedioxypyrovalerone (MDPV), which exert potent cocaine- or amphetamine-like properties and neurochemical actions, can also induce neuroinflammatory effects [10,11,12].

The ability of traditional and novel psychostimulants to affect neuroimmune signaling, as well as the degree to which they can do so, depends on many factors. These include psychostimulant type, dosage, duration of intake or exposure, time elapsed between drug clearance and tissue analysis, and the brain region or systemic bodily compartment analyzed [3,4,5]. Not surprisingly, results of studies on these phenomena can vary widely, posing difficulty in interpreting the overall impact of psychostimulants on neuroimmune signaling. For example, many studies in animals have examined neuroinflammatory markers in tissue or plasma after a relatively short amount of time following psychostimulant exposure or intake (<1 week) [11,13,14], whereas, in humans, plasma levels of inflammatory cytokines can be altered for months or even up to a year following cessation of drug use [15,16,17,18]. Another confounding factor in preclinical animal studies is whether the psychostimulant is administered passively by an experimenter (often in supraphysiological bolus doses), as compared to extended drug exposure under volitional control by the experimental subject. Such variations in drug administration methodology can lead to differences in drug pharmacokinetics, expectancy, and motivational state, thus influencing neuroimmune responses to psychostimulants. Such influences have been demonstrated in studies investigating other physiological effects of psychostimulant exposure (e.g., monoaminergic, neuroendocrine, cellular, molecular, structural, and functional changes) [19,20,21,22,23,24]. Similar modulatory effects of the locus of control on neuroimmune activation have been found in response to exposure to inescapable vs. escapable stressors [25]. Finally, sex differences in central markers of neuroinflammation in response to psychostimulants, particularly methamphetamine (METH) and synthetic cathinones, have been understudied, highlighting the need for further investigation of this topic [11,12].

Therefore, the present study was designed to address several of the aforementioned gaps in the literature. First, we sought to examine if changes in central neuroimmune markers induced by the psychostimulants METH and MDPV persist beyond the acute abstinence phase, particularly since persistent neuroinflammation may play a role in the intractability of drug intake and drug-seeking behavior. Building off our prior studies where we observed MDPV-induced impairments in cognitive function 21 days after cessation of drug intake [26], we chose a 3-week time point as representative of the post-acute abstinence phase. Second, since subgroups of psychostimulant users engage in binge-like patterns of intake over several days, interspersed by several days of abstinence [27,28,29,30,31], we employed a self-administration paradigm consisting of 96-h (4 days) drug access periods, each of which are followed by 72 h (3 days) of abstinence in the home cage. This process is repeated several times to model multiple binge-like episodes [12,26,32,33]. Third, we sought to observe potential persistent sex differences in central markers of neuroinflammation following binge, like METH or MDPV intake. For our analysis of central neuroimmune activation, we selected the prefrontal cortex (PFC) and dorsal striatum (DStr), which are heavily implicated in addictive processes and drug-induced disruption of cognitive function and are innervated by ascending monoaminergic systems that are the primary neuropharmacological targets of psychostimulants [1,2,3,4,5,6].

## 2. Materials and Methods

### 2.1. Animals

A total of n = 61 male and n = 65 female Sprague-Dawley rats (300–350 g upon arrival, Envigo, Placentia, CA, USA) were used in the current study. Prior to surgical procedures, animals were pair housed in a vivarium on a reversed light–dark cycle (12:12; lights off at 0700 h). Temperature and humidity levels were maintained within guidelines of the National Institutes of Health Guide for the Care and Use of Laboratory Animals (8th edition) [34]. Following implantation of intravenous catheters, rats were single housed to minimize potential damage to vascular access ports arising from cage-mate chewing. Food and water were always available ad libitum. All procedures were approved by the Institution Animal Care and Use Committee (IACUC) at Arizona State University, Tempe Campus.

### 2.2. Drugs

(+)-Methamphetamine hydrochloride was purchased from Sigma-Aldrich (St. Louis, MO, USA). Racemic MDPV was provided by the National Institute on Drug Abuse Drug Supply Program (Research Triangle Park, NC, USA). Drugs were dissolved in sterile 0.9% *w*/*v* sodium chloride for self-administration procedures.

### 2.3. Implantation of Intravenous Catheters

Rats were placed under anesthesia using isoflurane (5%) vaporized in oxygen at a flow rate of 2 L/min, and the isoflurane concentration was subsequently reduced to 2–3% during maintenance of anesthesia. After exposing the jugular vein and creating a small venous incision, silastic catheters were inserted ~3.0 cm into the vein and then secured in place with sutures. The opposite end of the catheter was tunneled subcutaneously to exit the skin between the scapulae and connected to a vascular access port (Instech Laboratories, Plymouth Meeting, PA, USA). Ports were secured to the surrounding skin with sutures and flushed with 0.2 mL of heparinized Timentin (66.6 mg/mL ticarcillin/clavulanic acid and 70 U/mL heparin in sterile saline) to minimize postsurgical infections. During the first 5 days following surgical procedures, daily infusions of heparinized Timentin solution were performed to maintain catheter patency, after which animals received daily infusions of heparinized saline. During the first 3 days of postsurgical care, rats were administered meloxicam (2 mg/kg s.c.) daily to minimize potential post-surgical discomfort. Animals of either sex were then randomly assigned to commence acquisition of intravenous self-administration of either METH or MDPV, or saline as a control. In order to verify catheter patency, the short-acting barbiturate sodium methohexital (Brevital, 10 mg/kg) was infused once weekly to ascertain periods of brief immobility.

### 2.4. Apparatus

Operant conditioning chambers (Med Associates, St. Albans, VT, USA) that were interfaced to a PC computer were used in these studies. Each chamber contained two nosepoke apertures (2.5 cm diameter), one designated the “active” aperture, and the other being designated the “inactive” aperture. A white stimulus light was positioned above the active aperture that would be illuminated following activation, along with simultaneous presentation of auditory tone (~65 dB, 2900 Hz) from a speaker placed near the top of the chamber and activation of a syringe pump. Responses on inactive pokes had no consequences (drug delivery, tone, and light) but were recorded to confirm that rats learned that responses on the active lever were responsible for drug delivery. Before and during each self-administration session, food pellets were placed on the floor of the chamber, and water was provided in a water bottle mounted on one chamber wall. Drug solutions (METH, MDPV, or saline) were delivered to the animal via a syringe located in a PC-controlled syringe pump that was connected with polyethylene (PE) tubing to a liquid swivel that was mounted above the chamber. A protective metal tether was attached to the swivel and housed PE tubing that was connected to the vascular port. Each chamber was housed separately in a sound-attenuating cubicle that also contained a house light programmed to turn on or off at the same time as in the colony room. Cubicles also contained ventilation fans to mask external noise and odors.

### 2.5. Self-Administration Procedures

**Figure 1:**
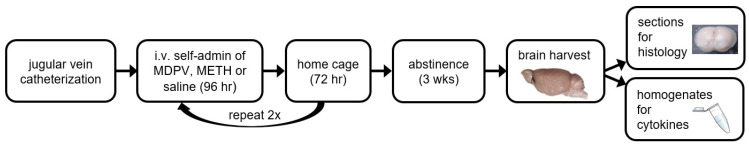
Design and experimental timeline of the current study.

Rats were placed in self-administration chambers without any prior training and were allowed to spontaneously acquire i.v. self-administration of METH, MDPV, or saline. Each session was 96 h in length. Doses of 0.05 mg/kg/infusion for METH and MDPV were selected based on our prior studies using both limited and 96-h binge-like access conditions where we observed robust self-administration in rats [12,26,33,35]. Based on drug dose and infusion volume, and depending on animal body weight, the resulting concentrations of METH and MDPV were 0.3–0.45 mg/mL. Following the first 96-h session, animals were placed into their home cage for 72 h of abstinence. This sequence was repeated twice, allowing each animal to undergo three 96-h self-administration sessions, each separated by 72 h of abstinence (see Figure 1) [26,32,33].

All i.v. infusions were delivered on a fixed-ratio 1 (FR1) schedule of reinforcement. Nosepokes into one aperture that was designated as the active aperture resulted in reinforcer delivery in a volume of 0.06 mL over a 1-s period, accompanied by concurrent activation of the stimulus light located above the aperture, and presentation of an auditory tone. Following each infusion, a 20-s timeout period was enacted, where additional active nosepokes were recorded but had no programmed effects. Nosepokes into a separate aperture designated as inactive produced no programmed consequences at any time. Prior to each session, 0.1 mL of heparinized saline solution was flushed through the catheter. This process also occurred daily during each 72 h of abstinence. For METH and MDPV self-administration, an acquisition criterion of at least 50 infusions obtained during the first 96-h session was imposed in order for animals to be included in subsequent analyses. Animals failing to meet these criteria were removed from the study. We elected not to utilize yoked saline administration as a control since the one of the primary purposes of the current study was to examine effects of voluntary drug intake, and prior studies have shown that response-independent delivery of drug or saline solutions can produce aversive properties and reduce psychostimulant intake [36]. Following the last 96-h session, rats were returned to the home cage, where they remained undisturbed for 3 weeks, with the exception of once-weekly cage changes. Following 3 weeks of abstinence, animals were euthanized, and brain tissue from each subgroup of animals for each sex and experimental group (METH, MDPV, or saline self-administration, n = 4–9/group) were randomly selected to undergo processing for either immunohistochemical, Golgi, or TUNEL staining (see Section 2.6, Section 2.7, Section 2.8, Section 2.9 and Section 2.10 below). At the same time point, additional subgroups of animals from each sex and experimental group (n = 4–6/group) were euthanized by CO_2_ asphyxiation, followed by rapid brain removal and processing for assessment of tissue homogenate cytokine levels (see Section 2.11 below).

### 2.6. Immunohistochemical Staining

Animals were deeply anesthetized with a euthanasia solution (Somnasol, Covetrus North America, Portland, ME, USA) and then perfused transcardially with ~200 mL of phosphate-buffered saline (PBS), followed by perfusion with ~250 mL of 4% *w*/*v* paraformaldehyde (PFA). Brains were then removed and stored in 4% PFA overnight at 4 °C. Coronal sections (100 μm thickness) containing the prelimbic and infralimbic cortices (collectively termed the PFC, +2.5 to +3.7 mm rostral to bregma as defined by the atlas of Paxinos and Watson [37]) or the DStr (+0.2 to +2.3 rostral to bregma) were collected on vibratome (VT1000S, Leica Microsystems, Deer Park, IL, USA) and stored in PBS at 4 °C.

For immunostaining, sections were washed 3 × 10 min in PBS and blocked in PBS containing 0.1% *v*/*v* Triton-X 100 (PBST) and 5% *v*/*v* normal donkey serum for 2 h. Tissue sections then underwent incubation with one of the following primary antisera (diluted in blocking solution) under gentle agitation overnight at 4 °C: goat anti-glial fibrillary acidic protein (GFAP, astrocyte marker, 1:1000, Abcam #ab53554, Boston, MA, USA), mouse anti-neuronal nuclei (NeuN, neuron marker, 1:1000, MilliporeSigma #MAB377, St. Louis, MO, USA), or goat anti-ionized calcium binding adaptor molecule 1 (Iba1, microglial marker, 1:500, Abcam #ab5076). Following primary antibody incubation, sections were then washed 3 × 10 min with PBST at room temperature. Next, tissue sections were incubated with either AlexaFluor647-conjugated donkey anti-goat IgG (Abcam #ab150135) or donkey anti-mouse IgG (Abcam #ab150107) secondary antisera at a dilution of 1:500 in blocking solution under gentle agitation for 2 h at room temperature. Next, sections were washed 3 × 10 min in PBST, followed by a final rinse 1 × 10 in PBS. Finally, sections were placed onto microscope slides. VectaShield antifade mounting medium (Vector Laboratories, Burlingame, CA, USA) was applied and overlaid with a coverslip and sealed with CoverGrip Coverslip Sealant (Biotium, Fremont, CA, USA), and then the slides were stored at 4 °C, protected from light, until analysis.

### 2.7. Quantification of Cell Density

A Zeiss LSM 710 confocal microscope (Carl Zeiss Microscopy, White Plains, NY, USA) was used to collect x-y tile scans of entire coronal sections at 633 nm excitation, 20× magnification, zoom factor of 1.0, scan speed 3.87 s per frame, and 0.42 µm pixel size. The resulting images (1024 × 1024 resolution) were imported into ImageJ/FIJI v. 1.54f, outlines of the PFC and DStr were drawn, and areas were measured in mm^2^. For assessment of astrocyte and microglial density, the numbers of GFAP or Iba1 labeled cells per mm^2^ were manually counted using the Multipoint tool in ImageJ/FIJI by investigators blind to sex and experimental group. For assessment of neuronal density, we utilized a semi-automated method described elsewhere [38]. Briefly, images were imported into ImageJ/FIJI by a blinded investigator, and a macro that executed the following functions was utilized: (1) automatic removal of excessively bright spots with radius > 2 pixels and pixel intensity > 200; (2) bandpass filter of objects > 61.2 µm and <4.6 µm in diameter; (3) autoscaling and saturation of pixel intensities to conserve intensity differences; (4) creation of a binary image using the Intermodes algorithm; (5) conversion to mask to create image consisting only of identified cells and elimination of all other pixels; (6) Watershed filter to ensure adjacent objects were counted as separate cells; and (7) quantification of cells via the Analyze Particles function. For all cell density analyses, values were obtained from 2–5 images per region and averaged within region across n = 5–8 animals per sex and experimental group.

### 2.8. Assessment of Microglial Morphology

Three-dimensional confocal images (z-stacks, n = 3–6 per region for each animal) of Iba1-stained issue were obtained at 633 nm excitation, 40× magnification, zoom factor of 0.8–1.0, scan speed of 7.75 s per frame, x-y pixel size of 0.13 µm, and a z-step size of 1 µm. Each z-stack (1024 × 1024 x-y resolution) was first preprocessed in ImageJ/FIJI, where lines were drawn perpendicularly through distal microglial process segments in order to generate histograms of pixel intensities to ascertain background pixel intensity values. Pixel intensities at or below this background level were then converted to background (i.e., black) in all images for that particular z-stack. Next, images were processed using 3DMorph (https://github.com/ElisaYork/3DMorph, accessed on 23 April 2024), a MATLAB-based script that allows for automated three-dimensional reconstruction of multiple microglia and quantification of various morphological parameters. These procedures were originally developed by York et al. [39] and have been implemented by others [40,41,42]. Briefly, analyses were performed in Interactive Mode, where thresholding and noise reductions were applied to further eliminate background without disconnection of process branches. Next, cells touching the border of the image field were eliminated as being incomplete, and then skeletonized reconstructions of individual microglia were generated and automatically analyzed for volume of three-dimensional space occupied by the cell (territorial volume) and to determine the ramification index (a measure of branching, calculated as territorial volume divided by volume of the microglia soma and all branches) and soma volume in μm^3^. Values were averaged across 3–6 sets of z-series images to form a single value of each parameter for each animal. Sample sizes of n = 5–8 animals were used for sex and experimental group.

### 2.9. Golgi–Cox Staining and Quantification of Dendritic Spine Density

For visualization of dendritic spines, we adapted methods published elsewhere for performing Golgi–Cox staining on thin sections [43,44]. We examined pyramidal neurons in layers 2/3 and layer 5 of the PFC and medium spiny neurons in the DStr, as prior studies have shown psychostimulant-induced alterations in dendritic spine density in these neuronal populations [45,46,47]. All procedures were performed with minimal exposure of the tissue and solutions to ambient light. Briefly, tissue sections were first immersed in a 1:1 mixture of Solutions A and B from a Rapid GolgiStain kit (FD Neurotechnologies #PK-410, Columbia, MD, USA) overnight at room temperature. On the following day, the solution was replaced with a freshly made amount of the same solution and stored at room temperature, protected from light, for 14 days, with brief gentle agitation performed twice weekly. Next, sections were placed in 1% *w*/*v* K_2_Cr_2_O_7_ for 30 min, mounted onto gelatin-coated slides, and then submerged in 28% *v*/*v* NH_4_OH for 30 min. Slides were then sequentially immersed in the following solutions: dH_2_O (5 min), developer solution (1:1 Solutions D and E from the Rapid GolgiStain kit in an equal volume of dH_2_O, 15 min), dH_2_O (5 min), 50% *w*/*v* ethanol (3 min), 70% *w*/*v* ethanol (3 min), 95% *w*/*v* ethanol (2 × 3 min), 100% *w*/*v* ethanol (2 × 3 min), and xylene (2 × 3 min). DePeX mounting was then applied to slides prior to adding coverslips, which were then sealed with CoverGrip Coverslip Sealant. Slides were stored at room temperature, protected from light, until analysis.

Z-stack image series of whole coronal sections were obtained under brightfield illumination on an Olympus 2000 VS200 slide scanner (Evident Technologies, Bethlehem, PA, USA) at 20× magnification, x-y pixel size of 0.27 µm, z-depth of 40 µm, and z-step size of 1 µm. Using Olympus OlyVIA v4.1 software, individual DStr medium spiny neurons and PFC pyramidal neurons with cell bodies located in layers 2/3 or layer 5 (n = 5–10 per region per animal) were identified, cropped, and exported as TIFF files (1024 × 1024 resolution). The resulting images were imported into ImageJ/FIJI, and dendrites of interest were manually traced, and their length was measured using the Segmented Line tool. For pyramidal neurons, only apical dendrites were analyzed, as we observed inconsistency in impregnation of basilar dendrites and their definitive connections with the cell soma, likely due to the use of thin sections. This also impaired our ability to assess overall dendritic arborization, and, thus, such analyses were not performed. Inclusion criteria for dendritic segments of PFC pyramidal neurons were localization to proximal portions of apical dendrites, >20 µm in length, being devoid of crossovers, and dendrites extending directly towards the cortical surface. Inclusion criteria for dendritic segments of DStr medium spiny neurons were segments > 25 µm in length, being devoid of crossovers, and localization beyond the first branch point since initial primary segments tend to be aspiny [48]. Numbers of dendritic spines in each segment were manually counted using the Multipoint tool. Dendritic spine density was calculated as the number of dendritic spines per 10 µm of dendritic branch length. All spine quantification was performed by an investigator blind to subject sex or experimental group.

### 2.10. Detection and Quantification of Apoptosis

To detect the presence of apoptotic cells, we first used a Click-iT Plus TUNEL Assay Kit for In Situ Apoptosis Detection (Alexa Fluor 488 dye, Invitrogen, Carlsbad, CA, USA) on coronal sections (100 µm thickness) according to the manufacturer’s directions. Sections were then incubated with a 1× solution of the far-red fluorescent nuclear stain RedDot2 (Biotium, Fremont, CA, USA) for 30 min; washed 1 × 10 min in PBS; coverslipped; sealed; and stored at 4 °C, protected from light, until analysis. Using a Zeiss LSM 710 confocal microscope, x-y tile scans of the PFC and DStr were obtained with sequential excitation at 488 and 633 nm at each frame, 20× magnification, scan speed of 1.94 s per frame, and pixel size of 0.42 µm. The resulting images (1024 × 1024 resolution) were then processed in ImageJ and cropped so that all image boundaries were within the PFC or DStr, and image channels were split into separate windows (TUNEL for green channel, nuclei for red channel). Further processing was performed as described by Abbass et al. [38]. For each green channel image, a fixed pixel intensity value of 80 was subtracted from all images as background; threshold minimum and maximum values were adjusted to 0 and 140, respectively; and a binary image was created which was despeckled and depixelated to reduce noise and smooth the image. For the red channel image, a fixed pixel intensity value of 80 was subtracted from all images as background; threshold minimum and maximum values were adjusted to 0 and 60, respectively; and a binary image was created. The Close function was used to fill any voids within nuclei, followed by the Outline function to create outlines around each nucleus. To remove small non-nuclei pixels, the Analyze Particles function was then used with a size range (in µm^2^) of 25.00 to infinity, circularity range 0.00–1.00, and outlines of nuclei touching the image edge were excluded. The processed green and red channel images were then merged, regions of analysis within the PFC or DStr were drawn, and areas were measured in µm^2^. Finally, the MultiPoint tool was used by an investigator blinded to sex or experimental condition to manually count outlines of nuclei that contained green (TUNEL) pixels. Green pixels outside of any identified nuclear boundary were ignored.

Given that our analyses revealed a decrease in Iba1+ cell densities in the PFC and DStr, we sought to confirm whether apoptotic (TUNEL+) cells were of microglial origin in additional tissue sections. Here, the immunohistochemical labeling of cells was performed as described in Section 2.6, with the exception that the antisera used consisted of a goat anti-Iba1 (as described above) and rabbit anti-cleaved (activated) caspase 3 (CC-3, 1:500, Cell Signaling Technology #9661, Danvers, MA, USA), and the secondary antisera consisted of AlexaFluor488-conjugated donkey anti-rabbit IgG and AlexaFluor568-conjugated donkey anti-goat IgG (diluted 1:500 in blocking solution). Following incubation with secondary antisera, sections were incubated with RedDot2, as described above, for visualization of cell nuclei. Images of triple-stained tissue in the PFC or DStr were obtained with a Zeiss LSM 710 confocal microscope with sequential excitation at 488, 564, and 633 nm at each frame; 20× magnification; a scan speed of 3.87 s per frame; and a pixel size of 0.42 µm. The resulting images (1024 × 1024 resolution) were then analyzed by an investigator blind to sex and experimental condition, and cells within the regions of interest that displayed pixel overlap from all 3 excitation wavelengths were counted and quantified as the number of cells per region.

### 2.11. Assessment of PFC and DStr Cytokine Levels

Animals were euthanized by CO_2_ asphyxiation, which was followed by rapid decapitation and removal of the brain. The reason for the use of this method over injectable or volatile anesthetic agents followed by transcardial perfusion and exsanguination is two-fold: (1) anesthetic agents can profoundly affect cellular mechanisms involved in cytokine production and signaling (i.e., activity extracellular signal-related kinase 1/2 and c-jun N-terminal kinase [49]); and (2) homogenates obtained from freshly dissected tissue show similar cytokine profiles as tissue obtained following transcardial perfusion and exsanguination, suggesting that cytokine levels in fresh tissue homogenates are primarily derived of brain origin and not from circulating plasma levels [50]. Dissected brains were rinsed with chilled PBS, and the PFC and DStr of each animal were dissected and placed separately into microcentrifuge tubes containing 0.5 mL chilled PBS with protease and phosphatase inhibitors. Tissues were homogenized with a sonicator and stored immediately at −80 °C. Total protein content of each sample was later determined by bicinchoninic acid (BCA) assay (ThermoFisher, Waltham, MA, USA), following the manufacturer’s directions. Frozen samples were diluted 1:1 in sterile PBS prior to shipping to Eve Technologies (Calgary, AB, Canada) for determination of levels of 27 different cytokines using a Rat Cytokine/Chemokine 27-Plex Discovery Assay^®^ (RD27). This assay utilizes Multiplexing LASER Bead Technology consisting of color-coded polystyrene beads conjugated to capture antibodies, and cytokine levels were detected using a Bio Plex 200 dual laser and flow cytometry system (Bio-Rad, Hercules, CA, USA). Quantification of individual cytokine levels was performed using 8-point sets of standards fitted with a cubic spline curve. The resulting values (in pg/mL) were then normalized to total protein levels of the respective sample to yield values expressed as ng/µg total protein.

### 2.12. Statistical Analyses

The number of animals excluded from analyses for various reasons is as follows: failure to meet acquisition criteria (n = 2), premature loss of catheter patency (n = 6), and loss of brain tissue integrity prior to histological analysis (n = 24). To analyze the number of METH, MDPV or saline infusions obtained during each 96-h session, a mixed-effects analysis of variance (ANOVA) with drug condition and 96-h session as between- and within-subjects factors, respectively, was performed, followed by Bonferroni’s correction for multiple comparisons. A similar mixed-effects ANOVA with drug condition and total infusions obtained across all three 96-h sessions as factors was also conducted, followed by Bonferroni’s multiple comparisons. For the analysis of cell densities, dendritic spine densities, microglial morphology, and number of apoptosis cells, separate two-way ANOVAs were performed for data from each brain region, with drug condition and sex as factors, followed by Tukey’s multiple comparisons tests. Normality for self-administration, cell and dendritic spine density, and co-localization data was verified using Shapiro–Wilk tests. For analysis of changes in cytokine levels, a separate multivariate ANOVA (MANOVA) was conducted for each brain region, followed by Bonferroni’s corrections for multiple comparisons. Levene’s tests for equality of variances were used to test for homogeneity of error for each cytokine analyzed. Principal components analysis (PCA) was also conducted to determine measures accounting for the cumulative proportion of variation observed. The lower cutoff value for a specific principal component to account for observed variance was set at 2%. All statistical analyses were performed using GraphPad Prism v.10.1 (GraphPad Software, La Jolla, CA, USA), except for MANOVAs, for which we utilized SPSS v.29 (IBM, Armonk, NY, USA). The *p*-values < 0.05 were considered statistically significant for all tests.

## 3. Results

### 3.1. Repeated 96-h Access to METH and MDPV Produces Robust Intake Levels

**Figure 2:**
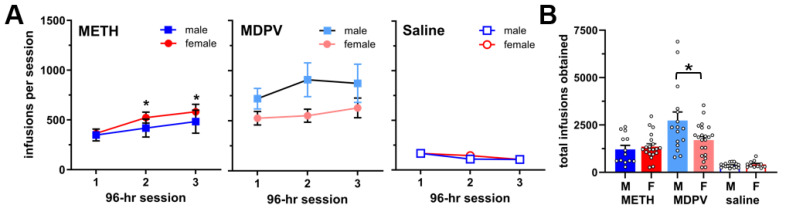
(**A**) Intake levels of METH (n = 13), MDPV (n = 16) and saline (n = 17) in male and female rats during each of three 96-h self-administration sessions. * *p* < 0.05 vs. session 1 for female animals. (**B**) Total number of infusions of METH, MDPV, or saline obtained across each of the three 96-h self-administration sessions. * *p* < 0.05 in males vs. females. All data are shown as mean ± SEM. Open circles represent data values from individual animals.

The significant main effects of drug condition (METH, MDPV, or saline, F_5,93_ = 11.48; *p* < 0.0001) and session (F_1.6,144.1_ = 4.43, *p* < 0.05) were observed, with animals obtaining significantly more METH and MDPV infusions than saline infusions. A drug condition × session interaction was not observed (F_10,179_ = 1.75, *p* > 0.05). Post hoc comparisons showed that METH intake was higher in females during sessions two and three as compared to the first session (Figure 2A; *p* < 0.05), but such effects were not observed in males (*p* > 0.05). For the analysis of the total number of infusions received across all three sessions, a main effect of drug condition (F_2,93_ = 30.14; *p* < 0.0001) and a sex × dug condition interaction (F_2,93_ = 4.20; *p* < 0.05) were observed, but there was no main effect of sex alone (F_1,93_ = 2.08; *p* > 0.05)_._ The post hoc comparisons revealed that total intake of METH and MDPV was higher than that of saline in both sexes (*p* < 0.05), but male animals showed significantly higher MDPV intake as compared to females (*p* < 0.05) (Figure 2B).

### 3.2. Astrocyte and Neuronal Density in the PFC and DStr Are Not Altered during Abstinence Following Repeated Binge-like METH and MDPV Intake

**Figure 3:**
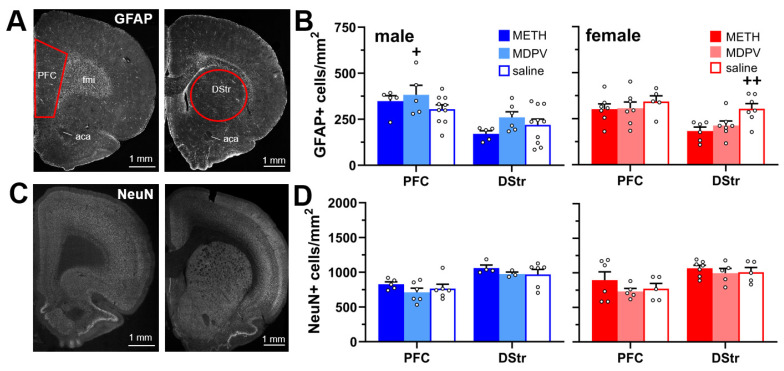
(**A**) Tiled confocal images of a representative coronal hemisphere stained for GFAP. Areas of PFC and DStr analyzed are outlined in red. *aca*, anterior commissure; *fmi*, forceps minor of corpus callosum. (**B**) Compared to saline controls (n = 5–10 per group), no changes in GFAP+ cell densities were observed in either region in METH (n = 5–7 per group) or MDPV (n = 5–7 per group) self-administering animals. However, GFAP cell density was higher in the PFC of male vs. female animals self-administering MDPV (+, *p* < 0.05), and GFAP cell densities in the DStr were higher in female vs. male saline controls (++, *p* < 0.05). (**C**) Tiled confocal images of a representative coronal hemisphere stained for NeuN. (**D**) Compared to saline controls (n = 5–6 per group), no changes in NeuN+ cell densities were observed in either region in METH (n = 4–7 per group) or MDPV (n = 3–6 per group) self-administering animals. All data are shown as mean ± SEM. Open circles represent data values from individual animals.

Our analysis of the number of GFAP-positive cells in the PFC and DStr (Figure 3A,B) revealed a significant effect of drug condition (F_5,68_ = 5.70, *p* < 0.0005) and a drug condition × sex interaction (F_5,68_ = 4.31, *p* < 0.005), but no main effect of sex (F_1,68_ = 0.10, *p* > 0.05). The post hoc comparisons revealed higher astrocyte density in the PFC of males vs. females self-administering MDPV (*p* < 0.05), and lower density in the DStr of males vs. females self-administering saline (*p* < 0.05). All other comparisons failed to reach statistical significance (*p* > 0.05).

In addition, the analysis of the number of NeuN-positive cells in the PFC and DStr (Figure 3C,D) revealed a significant effect of drug condition (F_5,51_ = 7.78, *p* < 0.0001) and no effect of sex (F_1,51_ = 0.42, *p* > 0.05) or a drug condition × sex interaction (F_5,51_ = 0.06, *p* > 0.05). The post hoc comparisons revealed that neuron density was lower in the PFC of male animals self-administering MDPV vs. the DStr of male animals self-administering METH (*p* < 0.05), the PFC of female animals self-administering MDPV vs. the DStr of females self-administering saline (*p* < 0.05), and the PFC of females self-administering saline vs. the DStr of females self-administering METH (*p* < 0.05). However, these comparisons were not deemed to be pertinent to the overall goal of the current study and were therefore excluded from further discussion. All other comparisons failed to reach significance (*p* > 0.05).

### 3.3. Dendritic Spine Densities in the PFC and DStr Are Reduced during Abstinence Following Repeated Binge-like Intake of METH and MDPV

**Figure 4:**
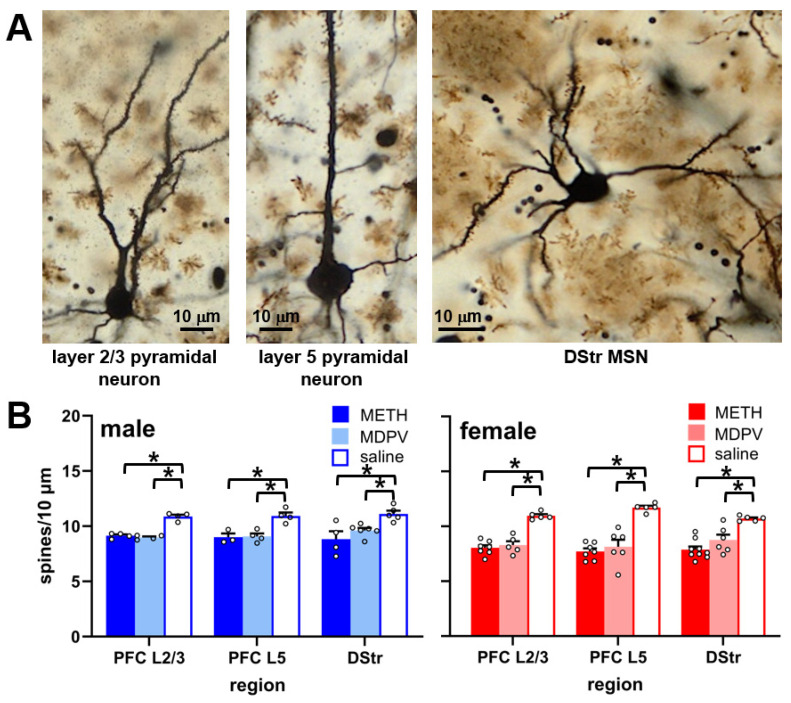
(**A**) Representative brightfield images of Golgi–Cox-impregnated neurons in layer 2/3 and layer 5 of the PFC, and a medium spiny neuron in the DStr. (**B**) Compared to saline controls (n = 5–6 per group), both male and female animals self-administering METH (n = 4–9 per group) or MDPV (n = 4–6 per group) showed decreased dendritic spine densities in all regions analyzed. * *p* < 0.05 vs. corresponding saline control group. All data are shown as mean ± SEM. Open circles represent data values from individual animals.

On apical dendrites of neurons located in layer 2/3 and layer 5 of the PFC, as well as in medium spiny neurons of the DStr (Figure 4A), we observed a significant effect of drug condition (F_8,79_ = 25.62, *p* < 0.0001) and sex (F_1,79_ = 12.05, *p* < 0.001), but not a drug condition × sex interaction (F_8,79_ = 1.93, *p* > 0.05). The post hoc comparisons revealed that, in both regions, dendritic spine densities were reduced in animals self-administering METH or MDPV as compared to animals self-administering saline, regardless of sex (Figure 4B). No sex differences in spine densities in either region were observed in saline self-administering animals (*p* > 0.05).

### 3.4. Abstinence Following Repeated Binge-like Intake of METH and MDPV Is Accompanied by Reduced PFC Microglial Cell Density and Evidence of Microglial Apoptosis

**Figure 5:**
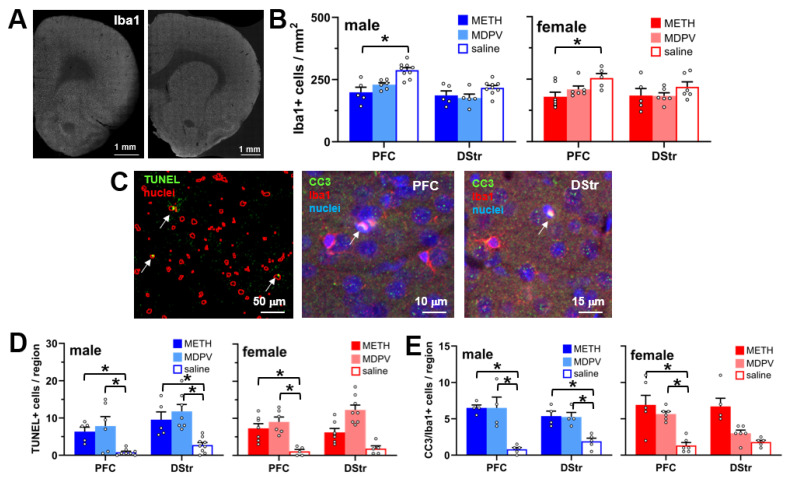
(**A**) Tiled confocal images of a representative coronal hemisphere stained for Iba1. (**B**) Compared to saline controls (n = 5–9 per group), decreased densities of Iba1+ cells were observed in the PFC of male and female animals self-administering METH (n = 5–7 per group). * *p* < 0.05 vs. saline. (**C**) *Left*, representative tissue section showing outlines of nuclei (red) stained by the far-red nuclear stain RedDot2, and TUNEL staining (green). Arrows highlight TUNEL+ nuclei. *Middle and right*, immunostaining of the PFC and DStr for the apoptosis marker cleaved caspase 3 (CC3, green) and microglial marker Iba1 (red), counterstained with nuclear marker RedDot2 (blue). Arrows denote co-localization of all three signals. (**D**) Quantification of TUNEL+ cells in the PFC and DStr of male and female rats self-administering METH (n = 5–7 per group), MDPV (n = 6–8 per group), or saline (n = 4–9 per group). * *p* < 0.05 vs. corresponding saline groups. (**E**) Quantification of co-localized CC3, Iba1, and nuclei in the PFC and DStr of male and female rats self-administering METH (n = 4–6 per group), MDPV (n = 4–6 per group), or saline (n = 5–6 per group). * *p* < 0.05 vs. corresponding saline groups. All data are shown as mean ± SEM. Open circles represent data values from individual animals.

The quantification of the number of Iba1-positive cells in the PFC and DStr (Figure 5A,B) revealed a significant effect of drug condition (F_5,61_ = 9.03, *p* < 0.0001) but no effect of sex (F_1,61_ = 1.29, *p* > 0.05) or a drug condition × sex interaction (F_5,61_ = 0.50, *p* > 0.05). Post hoc comparisons revealed that compared to animals self-administering saline, regardless of sex, animals self-administering METH showed reduced microglial cell density only in the PFC (*p* < 0.05). Using TUNEL methods for the detection of apoptosis (Figure 5C), we observed a significant effect of drug condition on the number of TUNEL-positive cells (F_5,66_ = 19.52, *p* < 0.0001) but no main effect of sex or a drug condition × sex interaction (all *p*’s > 0.05). Increased numbers of TUNEL-positive cells were observed in both the PFC and DStr of male METH and MDPV self-administering animals, and only in the PFC of female animals self-administering METH or MDPV (Figure 5D, *p* < 0.05). Since we observed reduced microglial cell density in METH and MDPV self-administering animals, as described above, we utilized immunohistochemistry to determine if apoptotic cells expressed the microglial cell marker Iba1 (Figure 5C). An analysis of the number of cells showing co-localization of Iba1 and CC3 revealed a significant effect of drug condition (F_5,48_ = 19.97, *p* < 0.0001) but no main effect of sex or a drug condition × sex interaction (all *p*’s > 0.05). Similar to patterns of TUNEL staining, increased numbers of Iba1/CC3-positive cells were observed in the PFC and DStr of male METH and MDPV self-administering animals, and only in the PFC of female METH or MDPV self-administering animals (Figure 5E, *p* > 0.05).

### 3.5. Evidence for Alterations in Microglial Morphology during Abstinence Following Repeated Binge-like Intake of METH and MDPV

**Figure 6:**
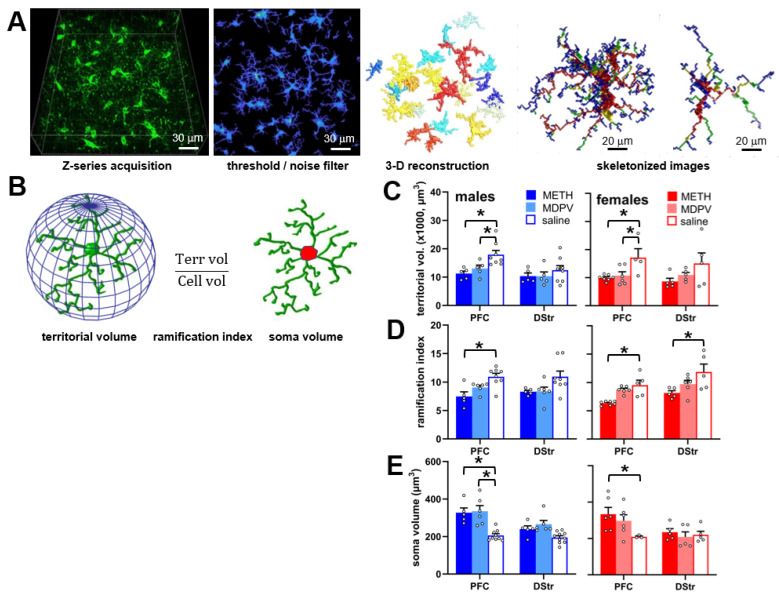
(**A**) Workflow for image processing and analyses of microglia morphology using 3DMorph. (**B**) Depiction of morphological parameters analyzed, where territorial volume is the volume of the 3D space occupied by a microglial cell and its branches, ramification index (territorial volume divided by cell volume, and soma volume (red). (**C**–**E**) Quantification of microglial territorial volume (**C**), ramification index (**D**), and soma volume (**E**) in the PFC and DStr of male and female animals self-administering METH (n = 5–7 per group), MDPV (n = 5–6 per group), or saline (n = 3–8 per group). * *p* < 0.05 corresponding saline controls. Open circles represent data values from individual animals.

An analysis of various measures of microglial morphology, which are indicative of inflammatory state, revealed a significant effect of drug condition on microglial territorial volume (F_5,55_ = 6.93, *p* < 0.0001), ramification index (F_5,60_ = 9.62, *p* < 0.0001), and soma volume (F_5,59_ = 10.85, *p* < 0.0001). No effects of sex or drug condition × sex interactions were observed for any of these measures (all *p*-values > 0.05). Compared to animals self-administering saline, microglia in the PFC but not DStr of males and females self-administering METH or MDPV showed reduced territorial volumes (Figure 6C; *p* < 0.05). The ramification index values were reduced in animals of both sexes self-administering METH compared to respective saline controls, and in the DStr, only microglia from female animals self-administering METH showed reduced ramification index values (Figure 6D; *p* < 0.05). Finally, compared to saline controls, male and female METH self-administering animals showed an increased microglial cell soma volume in the PFC, and only male MDPV self-administering animals showed an increased microglial cell soma volume in this region (Figure 6E; *p* < 0.05). The microglial cell soma volumes in the DStr were not different across sexes or drug conditions (Figure 6E; *p* > 0.05).

### 3.6. Abstinence Following Repeated Binge-like Intake of METH but Not MDPV Is Associated with Changes in Specific Cytokines in the PFC and DStr

**Figure 7:**
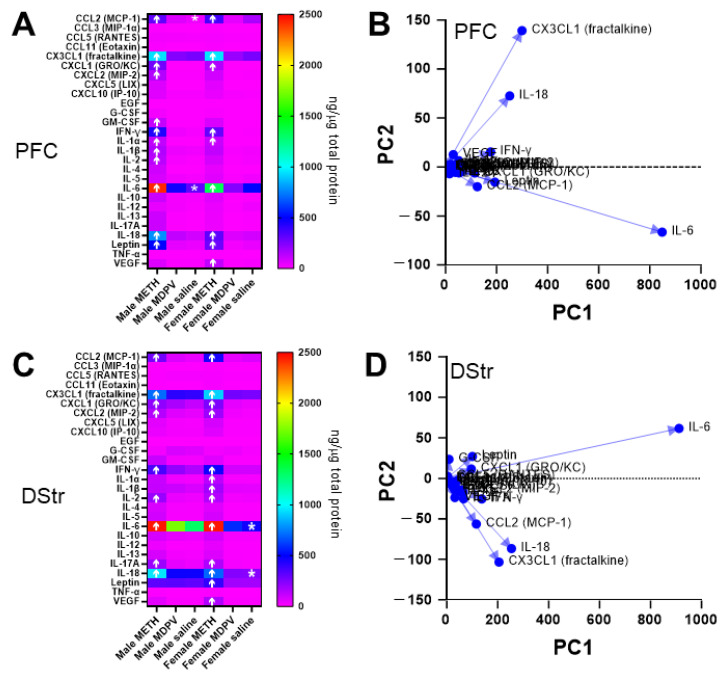
(**A**) Profile of changes in cytokines in the PFC in animals self-administering METH, MDPV, or saline. Arrows indicate significant (*p* < 0.05) increases or decreases as compared to respective saline groups. * Values in male animals self-administering saline that were significantly lower than those in corresponding female animals. (**B**) PCA loading plot showing primary cytokines in the PFC driving observed variances across experimental groups. (**C**) Profile of changes in cytokines in the DStr in animals self-administering METH, MDPV, or saline. Arrows indicate significant (*p* < 0.05) increases as compared to respective saline groups. * Values in female animals self-administering saline that were significantly higher than those in corresponding male animals. (**D**) PCA loading plot showing primary cytokines in the DStr driving observed variances across experimental groups. Samples sizes are n = 4–6 per sex and experimental group.

In the PFC, we observed a significant effect of sex (F_2,27_ = 113.18, *p* < 0.01) and drug condition (F_4,54_ = 51.31, *p* < 0.001), as well as a significant sex × drug condition interaction (F_4,54_ = 24.76, *p* < 0.01) on effects of METH and MDPV intake with regards to tissue cytokine levels. In comparison to male animals self-administering saline, male animals self-administering METH showed elevated levels of CCL2 (MCP-1), CX3CL1 (fractalkine), CXCL1 (GRO/KC), CXCL2 (MIP-2), GM-CSF, IFN-γ, IL-1α, IL-1β, IL-2, IL-6, IL-18, and leptin (Figure 7A; all *p*-values < 0.05). When compared to female animals self-administering saline, female animals self-administering METH showed increased levels of CCL2 (MCP-1), CX3CL1 (fractalkine), CXCL1 (GRO/KC), IFN-γ, IL-1α, IL-6, IL-18, leptin, and VEGF (Figure 7A; all *p*-values < 0.05). Surprisingly, no changes in any cytokine levels were observed in either male or female animals self-administering MDPV. In animals self-administering saline, we observed lower levels of CCL2 (MCP-1) and IL-6 in males compared to females, suggesting the existence of sex differences in basal levels of these cytokines (Figure 7A; all *p*-values < 0.05). The PCA analysis revealed a single principal component (PC1) that accounted for 94.79% of the variance, with an additional component (PC2) accounting for 2.9% of the variance (Figure 7B). Loading values for the primary cytokines that were observed to be altered (i.e., values > 100) were as follows (in rank order): IL-6 = 848.06, CX3CL1 (fractalkine) = 299.33, IL-18 = 251.21, leptin = 193.24, IFN-γ = 176.67, CCL2 (MCP-1) = 124.26, and CXCL1 (GRO/KC) = 114.02.

In the DStr, we found a significant effect of sex (F_2,27_ = 174.08, *p* < 0.01) and drug condition (F_4,54_ = 14.78, *p* < 0.01) and a significant sex × drug condition interaction (F_4,54_ = 12.24, *p* < 0.05) on effects of METH and MDPV intake with regards to tissue cytokine levels. Compared to males self-administering saline, male animals self-administering METH showed elevated levels of CCL2 (MCP-1), CX3CL1 (fractalkine), CXCL1 (GRO/KC), CXCL2 (MIP-2), IFN-γ, IL-2, IL-6, IL-17A, and IL-18 (Figure 7C; all *p*-values < 0.05). All of these cytokines were also elevated in the DStr of female animals self-administering METH, in addition to elevated levels of IL-1α, IL-1β, leptin, and VEGF (Figure 7C; all *p*-values < 0.05). Surprisingly, no changes in any cytokine levels were observed in either male or female animals self-administering MDPV. In animals self-administering saline, we observed lower levels of IL-6 and IL-18 in males compared to females, suggesting the existence of sex differences in the basal levels of these cytokines (Figure 7C; all *p*-values < 0.05). The PCA revealed PC1 that accounted for 94.18% of the variance, with an additional component (PC2) accounting for 2.8% of the variance (Figure 7D; all *p*-values < 0.05). PC1 loading values > 100 for the primary cytokines that were observed to be altered were as follows (in rank order): IL-6 = 911.71, IL-18 = 253.82, CX3CL1 (fractalkine) = 204.72, CCL2 (MCP-1) = 115.79, IFN-γ = 137.22, and leptin = 100.26.

## 4. Discussion

The 96-h binge-like self-administration paradigm, consisting of repeated periods of prolonged (96-h) drug access interspersed with 72 h of abstinence, was originally developed by Cornett and colleagues to provide a novel rodent model of multi-day binge and “crash” patterns of METH intake in humans [32]. Such access patterns typically result in high levels of METH intake (i.e., hundreds of infusions per session), which was observed in the present study. We observed that female rats escalated their intake over the course of several 96-h sessions, a result which has also been reported by others with this and other extended METH access paradigms [32,51]. This paradigm is also amenable to the study of binge-like patterns of intake of other psychostimulants, including the synthetic cathinone derivative alpha-pyrrolidinopropiophenone (α-PPP) and MDPV [12,26,33]. In the present study, MDPV intake was fairly robust, but also more variable, and we observed total MDPV intake to be higher in males vs. females. While various other studies have failed to find sex differences in MDPV intake [12,52], the differences observed here could be a result of different levels of R- and S-isomers in the stock MDPV used, as sex differences have been reported in MDPV isomers, as they relate to the steady-state volume of distribution, plasma protein binding, total clearance, and other pharmacokinetic parameters [53].

Many studies using either subchronic passive or active self-administration of METH result in astrogliosis when assessed within several days of cessation of drug exposure or access, as evidenced by increased density of GFAP-positive cells or overall GFAP immunoreactivity in various regions, including the PFC and DStr [54,55,56,57,58,59]. However, in the present study, we found no differences in astrocyte density in either the PFC or DStr at 3 weeks following binge-like intake of METH. One possible interpretation of these negative findings is that METH-induced astrogliosis may be transient in nature and resolved several weeks after discontinuation of METH exposure or intake, which may be reflected in subchronic and chronic administration paradigms used in other studies and the current study, respectively [54,55,56,57,58,59]. Additional studies using the 96-h access paradigm and assessment of astrocyte density at earlier time points would be necessary to establish this. It would also be of interest to examine changes in morphology of fine processes of astrocytic branches and their proximity to adjacent synapses which mediate neuron–glia crosstalk, but this usually requires transgenic or immunoelectron microscopy procedures due to the heterogeneity of astrocytic GFAP expression in some regions [60] and the need for viral vectors (i.e., AAV-Lck-GFP) for a full visualization of fine astrocytic process terminals [61]. We also observed no changes in DStr astrocyte density in either the PFC or DStr of animals self-administering MDPV, and with regards to the DStr, these effects are consistent with other studies showing no effect of subchronic passive MDPV exposure on astrocyte density as assessed 24 h following cessation of drug exposure [57,62].

Various prior studies have reported that high levels of METH intake or exposure induces neurodegeneration and neuronal loss in the PFC or DStr, as assessed by NeuN or other histological methods [58,59,63,64,65,66]. We have also reported MDPV-induced neurodegeneration in other regions, such as the perirhinal cortex [26]. It was therefore surprising that we failed to observe any changes in NeuN immunoreactivity in either of these regions following METH or MDPV intake. It is possible that the observed levels of METH or MDPV self-administered in the current study were insufficient to induce significant neurotoxicity. In addition, NeuN, a widely used non-specific neuronal marker, has been shown to be absent from some neuronal subpopulations in various brain regions, such as in cerebellar interneurons, olfactory mitral cells, and GABAergic neurons of the substantia nigra [67,68,69]. While we are not aware of any reports of cortical or dorsal striatal neurons lacking NeuN expression, the possibility still exists that METH or MDPV intake may have induced cytotoxic effects on some neuronal cell types in these regions, but not detected by quantification of NeuN immunoreactivity. However, in animals self-administering either METH or MDPV, we observed reduced densities of dendritic spines in apical dendrites of layer 2/3 and layer 5 PFC pyramidal neurons, as well as in DStr medium spiny neurons. Similar reductions in dendritic spine densities in these regions have been observed by other investigators during withdrawal from chronic passively or self-administered METH [45,46], as well as cocaine [47], which has neuropharmacological actions similar to those of MDPV. While different self-administration paradigms were used in these prior studies, necessitating caution when drawing direct comparisons to the present study, overall, these observations suggest that the repeated binge-like intake of METH and MDPV produces lasting reductions in the density of PFC and DStr dendritic spines, but not the neuronal number, as assessed by NeuN immunoreactivity.

Another surprising outcome of the present study was the observation that animals self-administering METH showed reduced microglial cell density in the PFC and DStr, as measured by quantification of Iba1 immunoreactivity, with no changes observed in MDPV self-administering animals. Based on prior studies, we anticipated observing increased microglial density induced by METH in one or both regions, as has been the case following acute withdrawal or abstinence from METH [56,59,70,71]. However, induction of inflammatory conditions and “activation” of microglia have been reported to cause irreversible transitions of microglia status towards cell death via various mechanisms, including apoptosis, necroptosis, ferroptosis, and pyroptosis [72]. We confirmed that the observed reductions in Iba1-positive cells in both the PFC and DStr were accompanied by apoptosis, as demonstrated by colocalization with TUNEL staining, as well as the apoptosis marker CC3 in these regions. However, these analyses also revealed TUNEL-positive cells in the DStr of animals self-administering METH, as well as in both the PFC and DStr in MDPV self-administering animals, which did not show reductions in microglial density. It therefore appears that non-microglial cells of an undetermined phenotype (e.g., endothelial, oligodendrocyte, etc.) also may have undergone apoptosis following MDPV intake. Additional further studies are warranted to confirm this. Yet, as expected, we observed strong evidence of sustained alterations in microglial cell morphology induced by METH, primarily in the PFC. Such changes included reduced microglial ramification and territorial volume and increase in cell soma volume, suggestive of transitions to inflammatory-like states of microglia [73]. Some of these effects, albeit to a more limited extent, were also observed in animals self-administering MDPV, which varied by region, sex, and specific morphological parameter. These observations confirm the induction of a persistent inflammation-like state in microglia, at least as determined by the morphological analysis, following the binge-like intake of METH and, to a more limited extent, MDPV. As it is now established that microglia mediate refinement at individual synapses throughout the lifespan [74,75], future investigations are needed to specifically determine whether METH- and MDPV-induced changes in microglial status are directly related to dendritic spine loss in the PFC and DStr.

When analyzing PFC tissue content for levels of various cytokines, we observed a number of alterations induced by METH. Namely, we observed elevated levels of CCL2 (MCP-1), CX3CL1 (fractalkine), CXCL1 (GRO/KC), IFN-γ, IL-1α, IL-6, and IL-18 in both male and female animals self-administering METH. Some sex differences were noted, such as elevations in CXCL2 (MIP-2), GM-CSF, IL-1β, and IL-2 in males only, whereas VEGF levels were elevated only in females. In the DStr, we observed increases in levels of CCL2 (MCP-1), CX3CL1 (fractalkine), CXCL1 (GRO/KC), CXCL2 (MIP-2), IFN-γ, IL-2, IL-6, IL-17A, and IL-18 in both male and female animals self-administering METH. Some sex differences were also noted in this region, with elevations in levels of IL-1α, IL-1β, leptin, and VEGF specifically in females. The principal components analysis revealed that the largest proportion of the variance observed (~94%, PC1 loading values > 100) was attributable to changes in IL-6, CX3CL1 (fractalkine), IL-18, leptin, IFN-γ, CCL2 (MCP-1), and CXCL1 (GRO/KC) in the PFC; and IL-6, IL-18, CX3CL1 (fractalkine), CCL2 (MCP-1), IFN-γ, and leptin in the DStr. These findings are consistent with previously published reports that have shown repeated short- or long-term METH administration to increase PFC and DStr tissue content of IL-1β and/or IL-6 ([56,76,77,78,79,80], though other studies have shown no such changes [81,82]. It is of note that, in other studies demonstrating results different than those observed here, animals did not undergo a period of abstinence following METH administration [81], or they underwent a series of behavioral tests following METH administration [82], which could partially explain these differences On the other hand, various reports indicate increases in levels of the pro-inflammatory cytokine TNF-α in the PFC and/or DStr following METH exposure ([56,77,80,81,83], yet we observed no changes in levels of this cytokine, perhaps because the time point for analysis was much later in the present study compared to previous studies. We also observed sex differences in levels of cytokines in saline self-administering animals, with increased levels of CCL-2 (MCP-1) and IL-6 in the PFC of females as compared to males, and lower levels of IL-6 and IL-18 in females vs. males, consistent with prior observations [84,85].

One of the most surprising observations of the present study was a complete absence of changes in brain cytokine levels in male or female animals self-administering MDPV. This is particularly puzzling in light of the number of other markers of inflammation observed in these animals (i.e., reduced microglial territorial volume and increased microglial cell soma volume), as well as some evidence of microglial apoptosis, though insufficient to cause a decrease in overall microglial density. Recently, Marusich et al. [11] reported that extended self-administration of α-pyrrolidinopentiophenone (α-PVP), a cathinone derivative with neuropharmacological actions similar to those of MDPV, in male rats produced increased levels of a variety of pro-inflammatory cytokines (i.e., IL-1α, IL-1β, IL-6, and CCL2 (MIP-2)) in the PFC and DStr. However, these analyses were performed within 24 h of the last drug intake session, and it is therefore possible that some of these changes may have also occurred in the present study with MDPV but abated during the 21-day period of abstinence. Indeed, in our previous study using capture antibody array methods to assess PFC levels of cytokines 21 days after drug self-administration in separate cohorts of animals [12], we did not observe changes in any of the cytokines contained in the current panel of analytes. Rather, we observed altered PFC levels of two other cytokines not analyzed in the current study, namely an increase in VCAM-1/CD106 and a decrease in Flt-3 ligand. Overall, it appears that MDPV, despite being readily self-administered and capable of producing persistent alterations in neuronal and microglial morphology, does not appear to induce a lasting state of neuroinflammation, as measured by tissue cytokine content.

The mechanisms by which METH induces neuroinflammation in forebrain regions such as the PFC, DStr, and other regions have been well-studied and include a multitude of events, including (but not limited to) compromised blood–brain barrier integrity leading to peripheral immune cell infiltration into the brain, direct activation of key immune signaling mediators such as astrocytic TNF-α secretion, excess glutamate release leading to excitotoxicity, and overproduction of cell-damaging cytotoxic reactive nitrogen or oxygen species. [1,2,3,4,5,6,81]. Far fewer studies have focused on examining the mechanisms by which synthetic cathinones such as MDPV cause neuroinflammation; however, many of the same mechanisms have been proposed, with additional possible contributions of overproduction of dopamine-derived quinones and mitochondrial dysfunction [10,86]. Interestingly, our findings of a lack of MDPV-induced gliosis are consistent with findings from other studies examining effects of different synthetic cathinones, such as mephedrone and methylone [86]. It is therefore apparent that, despite similar pharmacological mechanisms of action, synthetic cathinones differ greatly from classical psychostimulants such as METH and cocaine in their ability to evoke lasting inflammation in the brain.

While a detailed discussion of all observed changes in cytokine levels would be cumbersome, it is worth briefly discussing the main cytokine (IL-6) that was one of the primary drivers of group differences observed. In population-based studies, increased circulating IL-6 levels have been shown to be co-occur with cognitive decline [87], and patients with psychostimulant-use disorders who show high circulating levels of IL-6 perform significantly poorer on tests of executive function tests [88,89]. Changes in circulating levels of various cytokines, including IL-6, can persist for months or even up to a year following cessation of drug use [15,16,17,18]. Central neuroinflammatory adaptations such as increases in IL-6 and other cytokines in the PFC or DStr may induce maladaptive changes in corticostriatal circuit function that underlie aberrant synaptic plasticity, leading to persistent psychostimulant-associated memories, behavioral inflexibility, and poor self-control and decision-making, ultimately perpetuating the addiction cycle [7,90,91].

## 5. Conclusions

This study aimed to better understand the persistence of neuroimmune changes following psychostimulant use by analyzing the effects of repeated episodes of binge-like self-administration of METH and MDPV followed by 21 days of drug abstinence. Contrary to prior studies describing astrogliosis following subchronic passive or active METH self-administration, the present study found no changes in astrocyte density in the PFC or DStr, suggesting a possible transient nature of METH-induced astrogliosis that warrants further investigation under shorter periods of abstinence. Other notable findings of this study include the lack of evident PFC or DStr neuronal loss in METH or MDPV self-administering animals, as characterized by NeuN immunoreactivity. Despite this, changes in neuronal morphology were observed as reductions in PFC and DStr dendritic spine densities, suggesting lasting alterations in synaptic plasticity but not neuronal count. An analysis of microglial density following a 3-week abstinence from METH self-administering animals displayed a reduction in PFC and DStr microglial density, a surprising finding given subchronic passive or active models have previously shown increased microglial densities. Such reductions in microglia density, found to be a result of reactive apoptosis via TUNEL staining, confirm the induction of a persistent inflammatory state in microglia of these regions. Similar effects, albeit less pronounced, were found in animals self-administering MDPV, highlighting differential long-term effects between the psychostimulants. An analysis of changes in cytokine levels revealed drug-, region-, and sex-specific changes, suggesting complex interactions between the effects of psychostimulant intake, cellular and inflammatory responses, and the potential role of gonadal sex hormones. A striking finding was that MDPV intake did not result in persistent changes in tissue cytokine levels, despite evidence of alterations in microglial morphology and some evidence of microglial apoptosis. Collectively, our findings suggest that while both METH and MDPV share the common mechanism of increasing monoaminergic transmission in forebrain regions, there are clear differences in the lasting effects of these drugs on neuronal and glial morphology, as well as central inflammatory signaling. Nonetheless, these findings will help elucidate the persistent effects of repeated binge-like METH and MDPV effects on the brain, which may prove informative for neuroimmune-based treatment for psychostimulant-use disorders.

There are several limitations of the current study that require discussion. First, we chose to fill a critical gap in the literature examining more persistent neural, glial, and inflammatory effects of psychostimulant intake, as most studies examining these phenomena have examined brain tissue closer to the time of cessation of drug intake or exposure (i.e., within one week) [3,4,5]. While we tried to minimize potential external contributing factors by allowing the animals to remain undisturbed during the 3-week abstinence period, at this point, we cannot rule out the possible effects of weekly cage changes or other unknown environmental events. An additional limitation is the use of an FR1 schedule of reinforcement in the current study without assessing the possible effects of other fixed, variable, or progressive ratio paradigms. However, due to the complex nature of the current study, utilizing an FR1 schedule of reinforcement provided an initial starting point and large dataset that serves as a reference for other studies examining neuroinflammatory responses utilizing this common FR schedule. Other studies have reported influences of reinforcement schedule on self-administration patterns of both MDPV and METH [92,93], which in turn would alter the total amount of drug consumed and therefore potential neuroinflammatory or other cellular effects. However, to our knowledge, no direct explorations of the effects of reinforcement schedule on ability to alter central cytokine levels, microglial density and morphology, and dendritic spine density have been published, and this remains an important direction for future studies.

Another shortcoming of the present study was the examination of neuroinflammatory changes at one time point post-drug abstinence, given that changes in neuronal morphology, microglial status, and cytokine secretion are highly dynamic and occur on a much shorter timescale. In addition, our study utilized non-perfused brain tissue homogenates for cytokine analyses, making it possible for blood-derived cytokines to contribute to some observed effects. However, previous studies have found brain tissue cytokine concentrations to remain unaffected by brain exsanguination [44], suggesting that the observed changes are primarily reflective of brain tissue content levels. In addition, there are a number of other experimental variables and endpoints not analyzed in the present study, including estrus cycle phase of female animals, circulating levels of cytokines in plasma or serum, examination of specific subregions of the PFC and DStr where changes might have been more prominent (i.e., prelimbic and infralimbic cortices, dorsolateral vs. dorsomedial regions of the striatum), determining the type of apoptosis induced (i.e, necroptosis, ferroptosis, pyroptosis), and whether any effects observed are unique to the 96-h drug access paradigm used as opposed to more traditional extended access (i.e., 6 h/day) paradigms. All of these issues should be taken into consideration by investigators pursuing this line of inquiry.

## Data Availability

Original data from the described experiments will be made available to investigators upon request.
